# [^68^Ga]Ga-FAPI versus 2-[^18^F]FDG PET/CT in patients with autoimmune thyroiditis: a case control study

**DOI:** 10.1186/s13550-024-01129-y

**Published:** 2024-07-18

**Authors:** Kim M. Pabst, Lukas Kessler, Justin Ferdinandus, Rainer Hamacher, Timo Bartel, Jens T. Siveke, Michael Nader, Tim Brandenburg, Mélanie Desaulniers, Ken Herrmann, Wolfgang P. Fendler

**Affiliations:** 1 Department of Nuclear Medicine, West German Cancer Center, University Hospital Essen, Hufelandstr. 55, 45147 Essen, Germany; 2 German Cancer Consortium (DKTK), Partner Site University Hospital Essen, Essen, Germany; 3 Department of Radiology and Neuroradiology, University Hospital Essen, Essen, Germany; 4 Department I of Internal Medicine, Center for Integrated Oncology Aachen Bonn Cologne Duesseldorf, Medical Faculty and University Hospital Cologne, University of Cologne, Cologne, Germany; 5 Department of Medical Oncology, West German Cancer Center, University Hospital Essen, Essen, Germany; 6 Bridge Institute of Experimental Tumor Therapy, West German Cancer Center, University Hospital Essen, Essen, Germany; 7 Division of Solid Tumor Translational Oncology, German Cancer Research Center, DKFZ, Heidelberg, Germany; 8 Department of Endocrinology, Diabetes and Metabolism, Endocrine Tumor Center at West German Cancer Center, University Hospital Essen, Essen, Germany; 9 Endocrine Tumor Center at West German Cancer Center, Member of ENDO-ERN and EURACAN, University Hospital Essen, Essen, Germany; 10 Département de médecine nucléaire et radiobiologie, Université de Sherbrooke, Sherbrooke, QC Canada

## Abstract

**Purpose:**

Radiolabelled fibroblast activation protein inhibitors (FAPIs) are becoming increasingly important for imaging various tumour diseases. However, it is essential to be aware of potential pitfalls. Here, we investigate FAP expression in the thyroid gland in autoimmune thyroiditis (AIT).

**Methods:**

AIT patients with pathological thyroid uptake on [^68^Ga]Ga-FAPI PET were compared with glucose metabolism on 2-[^18^F]FDG PET in terms of SUV_max_/SUV_peak_/SUV_mean_/tissue-to-background ratio (TBR), and with a healthy control group.

**Results:**

Between September 2019 and July 2021, 6 patients presented with a visually increased thyroid uptake and TBR on [^68^Ga]Ga-FAPI PET. In the retrospective clinical work-up, all patients had known or newly diagnosed AIT. Compared to a matched healthy control group, FAP expression and glucose metabolism were significantly increased ([^68^Ga]Ga-FAPI (SUV_peak_): 7.0 vs. 1.7; *p* = 0.004/(TBR_bloodpool_): 6.8 vs. 1.7; *p* = 0.002; 2-[^18^F]FDG (SUV_peak_): 3.9 vs. 1.4; *p* = 0.004/(TBR_bloodpool_): 4.0 vs. 1.2; *p* = 0.041). However, there was no significant difference in median uptake between [^68^Ga]Ga-FAPI and 2-[18F]FDG PET (SUV_peak_: 7.3 vs. 5.6; *p* = 0.104).

**Conclusion:**

Patients with AIT show higher thyroid uptake on [^68^Ga]Ga-FAPI and 2-[^18^F]FDG PET. Incidental thyroid uptake is another pitfall in the interpretation of [^68^Ga]Ga-FAPI PET and should prompt a clinical work-up.

**Supplementary Information:**

The online version contains supplementary material available at 10.1186/s13550-024-01129-y.

## Introduction

Fibroblast activation protein (FAP) is expressed in the stroma of approximately 90% of all epithelial cancers, and is associated with their angiogenesis, migration and proliferation [1]. Its expression can be visualised using radiolabelled FAP inhibitors (FAPIs) on positron emission tomography/computed tomography (PET/CT) scans [2]. Despite improvements in FAP-directed radioligands [1, 2], false-positive uptake may occur in various conditions including acute or chronic inflammation, degenerative lesions and scarring [3]. In particular, inflammatory diseases such as pancreatitis and arthritis should be highlighted [4, 5], raising the question of whether [^68^Ga]Ga-FAPI PET/CT may also detect autoimmune thyroiditis (AIT) [6, 7], as fibroblast activation plays a role in all three diseases. In clinical practice, AIT is typically diagnosed by clinical symptoms, laboratory parameters and ultrasound [8].

The study aimed to retrospectively evaluate thyroid uptake on [^68^Ga]Ga-FAPI PET/CT using several semiquantitative parameters in six patients diagnosed with AIT and to compare the results with thyroid uptake on 2-deoxy-2-[^18^F] fluoro-d-glucose (FDG) PET/CT in order to better evaluate this pitfall in image analysis in the future.

## Materials and methods

**Fig. 1:**
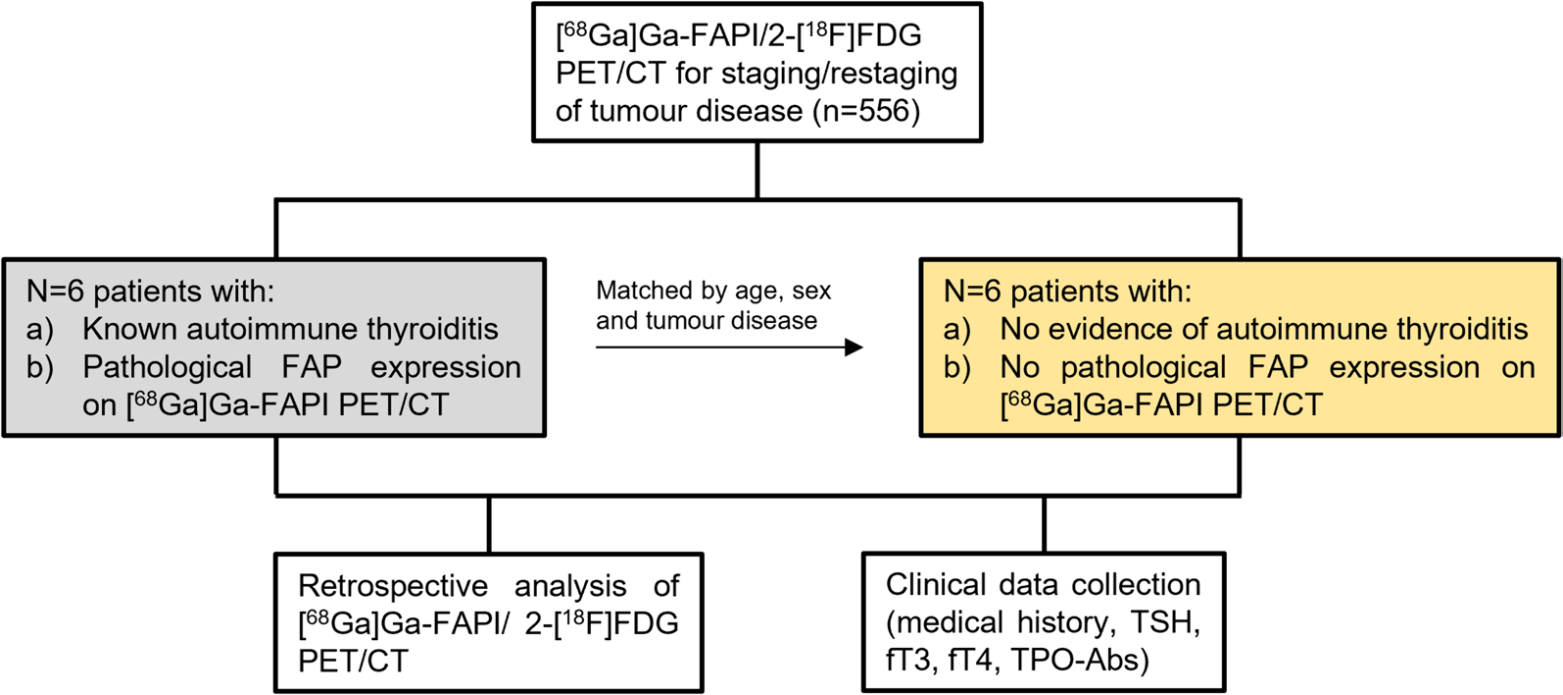
Study flow chart. Abbreviations: TSH: thyroid-stimulating hormone, fT3: free triiodothyronine, fT4: free thyroxine, TPO-Abs: thyroperoxidase antibodies

The patient flow is shown in Fig. 1. This subgroup analysis is part of the ongoing observational study (NCT04571086) at University Hospital Essen. Between September 2019 and July 2021, six patients diagnosed with AIT underwent [^68^Ga]Ga-FAPI PET/CT for complex oncological diagnoses and provided informed consent. Inclusion criteria were (a) [^68^Ga]Ga-FAPI PET/CT for tumour staging/restaging, (b) high thyroid uptake on visual assessment of [^68^Ga]Ga-FAPI PET and (c) age ≥ 18 years. 5/6 patients underwent additional 2-[^18^F]FDG PET/CT. Confirmation of AIT involved laboratory parameters (TSH, fT3, fT4, TPO-Abs), ultrasound findings, and medical history.

A group of six age-, sex- and disease-matched individuals without AIT at the time of [^68^Ga]Ga-FAPI/2-[^18^F]FDG PET imaging was assembled for comparison.

The radioligands used were [^68^Ga]Ga-FAPI-46 (n = 11) and [^68^Ga]Ga-FAPI-04 (n = 1). Radiosynthesis of [^68^Ga]Ga-FAPI-46 has been described previously [9]. Patients did not require fasting or special preparation. The median activity administered intravenously was 109 MBq (interquartile range (IQR): 72–144 MBq), with a median time from injection to acquisition of 18.5 min (IQR 10–77 min).

A 2-[^18^F]FDG PET/CT was performed in 11/12 (91.7%) patients, with a median administered activity of 270 MBq (IQR: 219–320 MBq); the median time from injection to acquisition was 72 min (IQR: 71–75 min). Diagnostic CT was performed and contrast was administered intravenously in 6/11 patients in accordance with current guidelines [10]. All PET scans were performed on a PET/CT system (Biograph mCT or Vision, Siemens, Erlangen, Germany).

SUV parameters including SUV_max_ (maximum standardised uptake value), SUV_mean_ (mean standardised uptake value), SUV_peak_ (peak standardised uptake value) were calculated with volumes of interest (VOIs) for both radioligands ([^68^Ga]Ga-FAPI and 2-[^18^F]FDG) using Syngo.via software (Siemens Healthineers, Erlangen, Germany) at 41% isocontour. Non-specific background noise in the mediastinal bloodpool (aortic vessel), liver and left gluteal muscle was quantified using a 2 cm diameter circular sphere to measure Tissue-to-Background ratios (TBR).

Descriptive statistics and individual patient data are reported. Statistical analyses were performed using GraphPad Prism (version 9.1.0; GraphPad Software, San Diego, California, USA) and SPSS (SPSS Statistics version 27.0, IBM, Armonk, New York, USA). SUV_max_/SUV_mean_/SUV_peak_ values for [^68^Ga]Ga-FAPI and 2-[^18^F]FDG PET were compared using the Wilcoxon test. Mann–Whitney-U test was performed to compare the diseased cohort with the healthy reference group. This retrospective analysis was approved by the local ethics committee (permits no. 20-9485-BO/20-9777-BO).

## Results

Six female patients with AIT and pathological FAP expression in their thyroid glands on [^68^Ga]Ga-FAPI PET/CT and six female controls were reviewed. The median age of the diseased population was 56 years (range 33–74 years), and for the control group, it was 57 years (range 39–73 years).

**Fig. 2:**
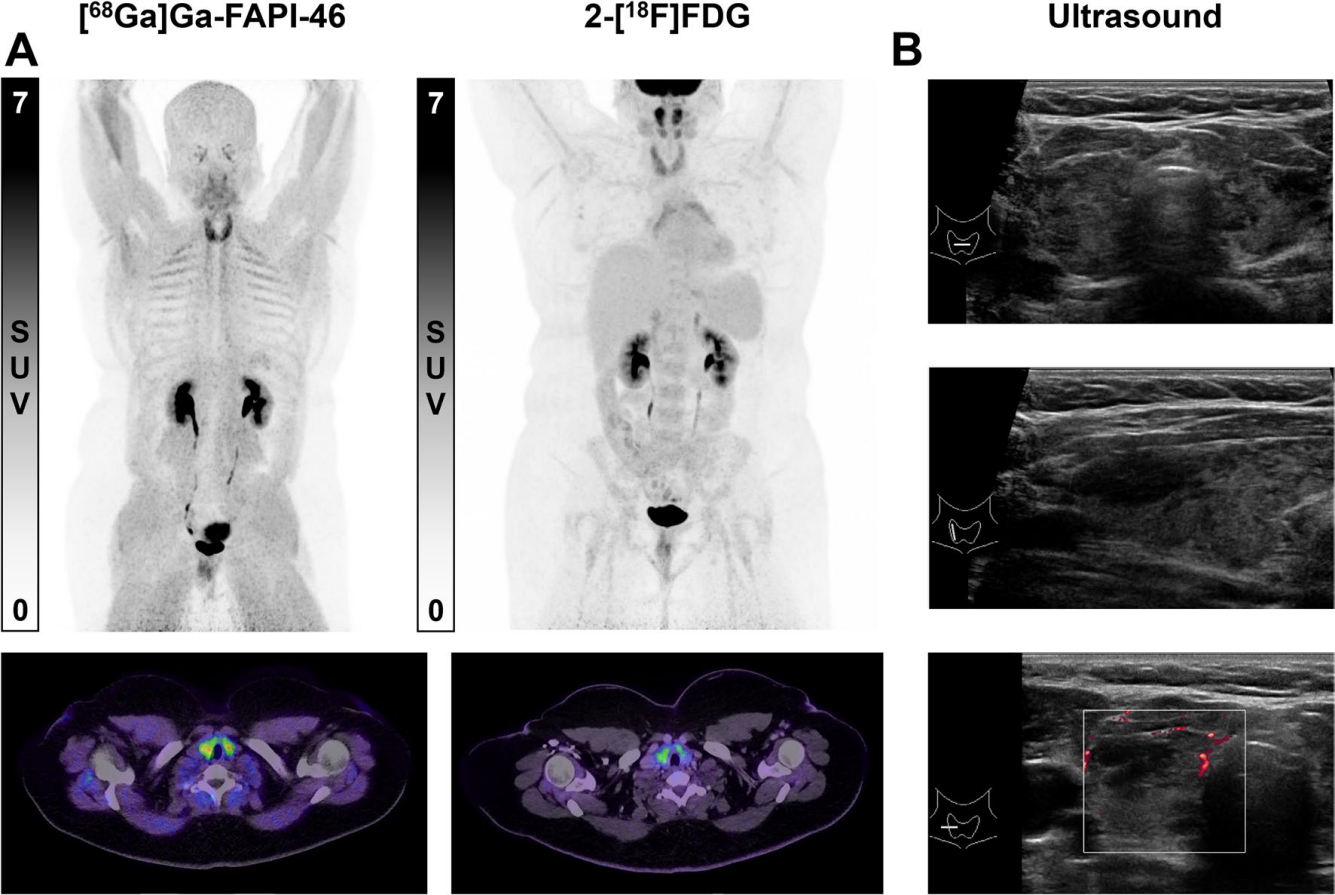
Example of thyroid uptake on [^68^Ga]Ga-FAPI-46 and 2-[^18^F]FDG PET in patient no. 3. **A** shows the maximum intensity projection of [^68^Ga]Ga-FAPI-46 and 2-[^18^F]FDG PET/CT and axial images of the thyroid. **B** shows ultrasound findings of the thyroid with inhomogeneous parenchyma and normal perfusion

5/6 (83.3%) AIT patients and 6/6 (100%) healthy controls underwent 2-[^18^F]FDG PET/CT. The median interval between both PET/CT scans was 0 days (range 0–8 days) for the AIT group and 0 days (range 0–2 days) in the control group. Imaging results of patient no. 3 are shown in Fig. 2.

All patients had known (n = 5) or newly diagnosed (n = 1) AIT. The median interval between [^68^Ga]Ga-FAPI PET/CT and laboratory parameter assessment was 60 days (IQR: 25–68 days). Table 1 provides further details. Two AIT patients underwent ultrasound showing typical signs of chronic thyroiditis (inhomogeneous thyroid parenchyma, normal perfusion). 5 patients received thyroid replacement therapy.

**Table 1:** Patient characteristics

Patient no.	Age	Gender	Diagnosis	TSH (mU/l)	fT3 (pmol/l)	fT4 (pmol/l)	TSH-R-Abs (IU/l)	TPO-Abs (IU/ml)	Tg-Abs (IU/ml)	Ultrasound	Thyroid medication
1	56	Female	SFT	4.8	4.8	19.9	1.13	> 1000	> 3000	–	l-Thyroxine 150 µg
2	55	Female	PDAC	1.54	–	–	–	–	–	–	l-Thyroxine 50 µg
3	33	Female	Endometrial stroma sarcoma	4.31	4.7	15.5	< 0.80	658	876	Inhomogenous, normal perfusion	l-Thyroxine 100 µg
4	41	Female	UPS	1.56	4.7	14.8	< 0.80	681	27	Inhomogenous, normal perfusion	Prothyrid 100 µg/10 µg
5	74	Female	PDAC	0.61	–	–	–	–	–	–	l-Thyroxine 100 µg
6	60	Female	Adenocarcinoma of the uterus	8.47	2.8	9.1	0.82	720	< 20	–	–
Median (IQR)	56 (45–59)	–	–	2.9 (1.5–4.7)	4.7 (4.2–4.7)	15.2 (13.4–16.6)	0.81	689	452	–	–

Patient with initial diagnosis of hypothyroidism marked in orange. *TSH* thyroid-stimulating hormone, *fT3* free triiodothyronine, *fT4* free thyroxine, *TSH-R-Ab* TSH-receptor antibodies, *TPO-Abs* thyroperoxidase antibodies, *Tg-Abs* thyroglobulin antibodies, *PDAC* pancreatic ductal adenocarcinoma, *SFT* solitary fibrous tumour, *UPS* undifferentiated pleomorphic sarcoma

Comparison of semiquantitative parameters (SUV_max_, SUV_mean_, SUV_peak_, TBRs) for thyroid uptake in AIT patients on [^68^Ga]Ga-FAPI and 2-[^18^F]FDG PET revealed no significant differences (median SUV_max_: [^68^Ga]Ga-FAPI: 9.6 (IQR: 8.4–10.4) vs. 2-[^18^F]FDG: 7.2 (IQR: 4.2–9.0), *p* = 0.144; median SUV_peak_: [^68^Ga]Ga-FAPI: 7.3 (IQR: 6.2–8.2) vs. 2-[^18^F]FDG: 5.6 (IQR: 2.9–6.5), *p* = 0.104; median SUV_mean_: [^68^Ga]Ga-FAPI: 5.3 (IQR: 4.8–6.1) vs. 2-[^18^F]FDG: 4.0 (IQR: 2.7–5.0), *p* = 0.176), except for TBR_bloodpool_/TBR_liver_. ([^68^Ga]Ga-FAPI vs. 2-[^18^F]FDG: TBR_bloodpool_: 6.8 vs. 4.0, p = 0.043; TBR_liver_: 12.5 vs. 3.4, *p* = 0.043; TBR_muscle_: 6.7 vs. 8.4, *p* = 0.893).

**Fig. 3:**
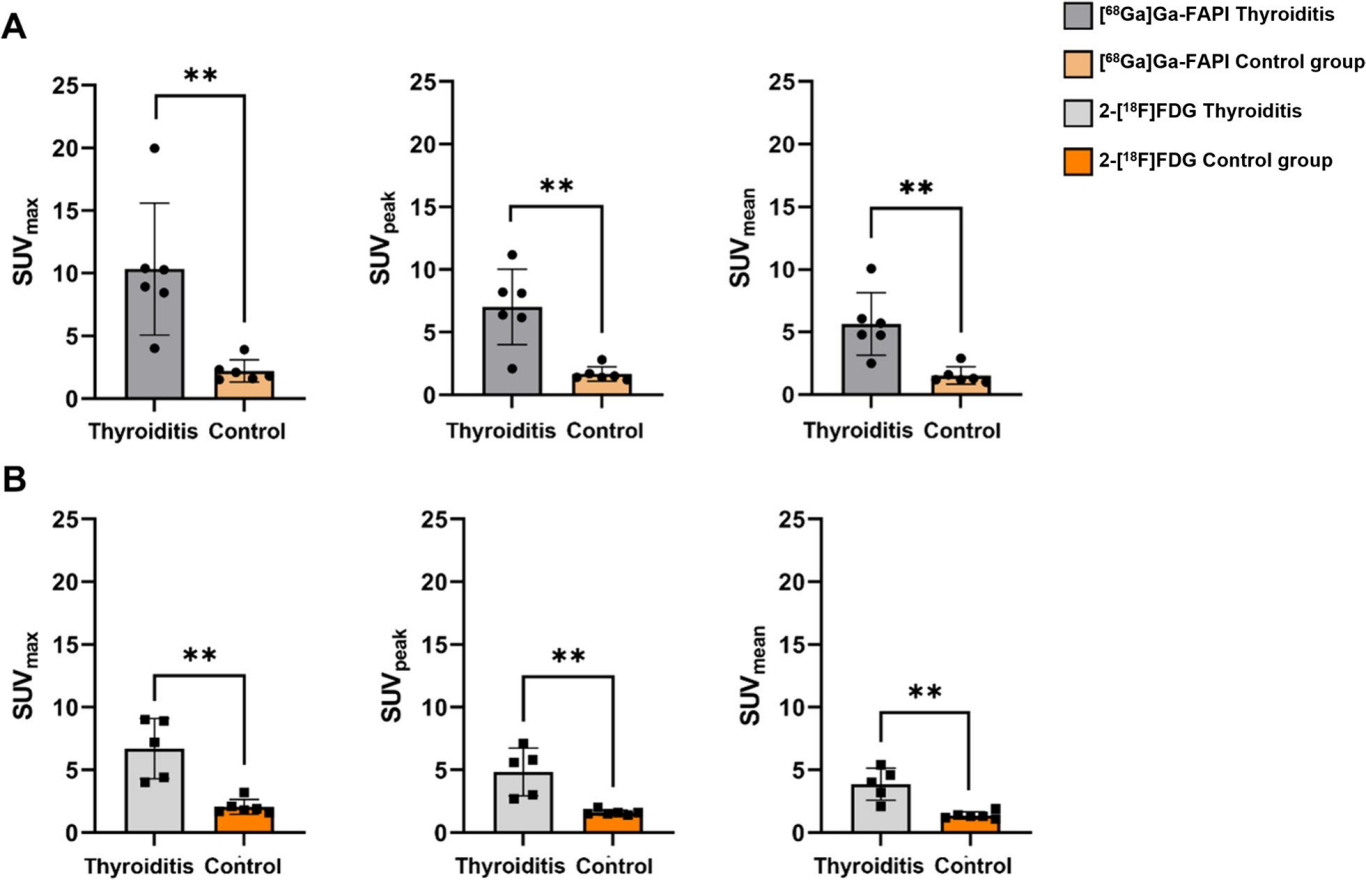
Comparison of thyroid uptake on [^68^Ga]Ga-FAPI and 2-[^18^F]FDG PET in patients with AIT and a healthy control group. **A** presents the comparison of SUV_max_/SUV_peak_/SUV_mean_ (median/standard deviation) values of AIT patients and a healthy control group for [^68^Ga]Ga-FAPI PET. **B** shows the corresponding 2-[^18^F]FDG PET results

All semiquantitative parameters examined on [^68^Ga]Ga-FAPI PET were significantly higher in AIT patients compared to the healthy controls (SUV_max_ (10.3 vs. 2.2; *p* = 0.002)/SUV_peak_ (7.0 vs. 1.7; *p* = 0.004)/SUV_mean_ (5.7 vs. 1.5; *p* = 0.004)/TBR_bloodpool_ (6.8 vs. 1.7; *p* = 0.002)/TBR_liver_ (12.5 vs. 3.3; *p* = 0.002)/TBR_muscle_ (6.7 vs. 1.4; *p* = 0.002). These results were comparable on 2-[^18^F]FDG PET (SUV_max_ (6.7 vs. 2.1; *p* = 0.004)/SUV_peak_ (3.9 vs. 1.4; *p* = 0.004)/SUV_mean_ (4.8 vs. 1.6; *p* = 0.004/TBR_bloodpool_ (4.0 vs. 1.2; *p* = 0.041)/TBR_liver_ (3.4 vs. 0.9; p = 0.002)/TBR_muscle_ (8.4 vs. 2.8; p = 0.009). A summary of patient characteristics is given in Fig. 3. Individual SUV values (SUV_max_/SUV_mean_) are shown in Additional file 1: Table S1.

## Discussion

Our retrospective analysis demonstrated a significant difference in thyroid uptake and TBR on [^68^Ga]Ga-FAPI PET in patients with AIT, in line could also be identified for 2-[^18^F]FDG, compared to a healthy control group. AIT is a pitfall for both 2-[^18^F]FDG [11] and [^68^Ga]Ga-FAPI PET.

The intense thyroid uptake in both imaging modalities is probably due to different mechanisms: While [^68^Ga]Ga-FAPI PET primarily represents fibroblasts and thus the fibrotic remodelling processes occurring in AIT [12], comparable to arthritis and pancreatitis [4, 5], the increased glucose metabolism on 2-[^18^F]FDG PET primarily represents inflammation. The superior delineation on [^68^Ga]Ga-FAPI PET may be attributed to lower background activity.

AIT progresses in several phases, from inflammation to fibrotic processes and scarring. Whether FAP expression differs between these phases and histopathological subtypes of AIT, particularly the fibrous variant, remains an open question. Various patterns of stromal fibrosis have been described, including interfollicular, interlobular, and scar fibrosis [13], which may contribute to the observed variance in FAP expression (SUV_max_ values ranging from 4.0 to 20.0) in our study. It is noteworthy that the sole patient with newly diagnosed AIT showed markedly higher SUV values in comparison to patients with previously known AIT (Additional file 1: Table S1). However, further data is required to prove a potential correlation.

Although our study has limitations, notably the retrospective design and a small patient cohort, it highlights significant differences in FAP expression and glucose metabolism in AIT patients compared to healthy controls. Further research, especially in potential subgroups of AIT, is warranted.

## Conclusion

Incidental thyroid uptake is another pitfall in the interpretation of [^68^Ga]Ga-FAPI PET, and also of 2-[^18^F]FDG PET. If thyroid uptake is high, additional testing should be performed to avoid misinterpretation.

### Supplementary Information

**Additional file 1: Supplemental Table 1.** Patient-based SUV values of the thyroid gland on [^68^Ga]Ga-FAPI and 2-[^18^F]FDG PET/CT.

## Data Availability

The datasets used and/or analysed during the current study are available from the corresponding author on reasonable request.

## Abbreviations

AIT: Autoimmune thyroiditis
FAPI: Fibroblast activation protein inhibitor
FDG: 2-deoxy-2-[^18^F] fluoro-d-glucose
fT3: Free triiodothyronine
fT4: Free thyroxine
PDAC: Pancreatic ductal adenocarcinoma
PET/CT: Positron emission tomography/computed tomography
SFT: Solitary fibrous tumor
SUV: Standardised uptake value
TBR: Tissue-to-background ratio
Tg-Abs: Thyroglobulin antibodies
TPO-Abs: Thyroperoxidase antibodies
TSH: Thyroid-stimulating hormone
TSH-R-Abs: TSH receptor antibodies
UPS: Undifferentiated pleomorphic sarcoma
