# Supplementary material for: [68Ga]Ga-FAPI versus 2-[18F]FDG PET/CT in patients with autoimmune thyroiditis: a case control study
Source: EJNMMI Res. 2024 Jul 18;14:66. doi: 10.1186/s13550-024-01129-y (PMC11258103; doi:10.1186/s13550-024-01129-y)
Supplement: Supplementary file 1 — Additional file 1: Supplemental Table 1. Patient-based SUV values of the thyroid gland on [68Ga]Ga-FAPI and 2-[18F]FDG PET/CT. [file 13550_2024_1129_MOESM1_ESM.docx]

**Supplemental Table 1: Patient-based SUV values of the thyroid gland on [^68^Ga]Ga-FAPI and 2-[^18^F]FDG PET/CT**

| **Patient No.** | **SUV_max_/ SUV_mean_ [^68^Ga]Ga-FAPI** | **SUV_max_/ SUV_mean_ 2-[^18^F]FDG** |
| --- | --- | --- |
| **1** | 10.3/6.1 | 9.0/5.4 |
| **2** | 8.9/4.8 | 8.9/4.6 |
| **3** | 10.4/5.7 | 7.2/4.0 |
| **4** | 4.0/2.5 | 4.4/3.2 |
| **5** | 8.4/4.8 | N.a. |
| **6** | 20.0/10.1 | 4.0/2.1 |
| **Median (IQR)** | 9.6/5.3  (8.6-10.4)/(4.8-6.0) | 7.2/4.0  (4.4-8.9)/(3.2-4.6) |

Patient with initial diagnosis of hypothyroidism marked in orange. Abbreviations: IQR: interquartile range. N.a.: not applicable.
